# Neuroradiological Findings in Cerebral Amyloid Angiopathy with a Particular Consideration of the Boston Criteria 2.0: An Imaging Review

**DOI:** 10.3390/biom14111459

**Published:** 2024-11-17

**Authors:** Ulf Jensen-Kondering, Katharina Heß, Alexander Neumann, Nils G. Margraf

**Affiliations:** 1 Department of Neuroradiology, University Medical Centre Schleswig-Holstein, Campus Lübeck, 23538 Lübeck, Germany; alexander.neumann@uksh.de; 2 Department of Radiology and Neuroradiology, University Medical Centre Schleswig-Holstein, Campus Kiel, 24105 Kiel, Germany; 3 Department of Pathology, University Medical Centre Schleswig-Holstein, Campus Kiel, 24105 Kiel, Germany; 4 Department of Neurology, University Medical Centre Schleswig-Holstein, Campus Kiel, 24105 Kiel, Germany

**Keywords:** cerebral amyloid angiopathy, Boston Criteria, neuroimaging, MRI, CT

## Abstract

In the elderly, cerebral amyloid angiopathy (CAA) is the most common cause for intracranial lobar hemorrhages. CAA is caused by the accumulation of amyloid-β fibrils in cortical and leptomeningeal vessels. In 2022, the Boston Criteria 2.0 became the new diagnostic standard for CAA, following the Modified Boston Criteria of 2010. The diagnostic criteria are a composite of clinical, imaging and histopathological findings. In the latest version of the Boston Criteria, neuroradiological imaging findings were even expanded compared to the previous version. Crucially, the correct application of the diagnostic criteria is necessary to avoid over- and underdiagnosis. The aim of this review is to demonstrate the diagnostic criteria for CAA with an emphasis on typical imaging findings which are part of the Boston Criteria 2.0 and other imaging findings suggestive of CAA.

## 1. Background

In the elderly, spontaneous cerebral amyloid angiopathy (CAA) is the most common cause of intracranial lobar hemorrhages [1]. Since recurrence risk is sevenfold higher than that in hypertensive intracranial hemorrhages, an accurate and prompt diagnosis is crucial. The clarification of the diagnosis of CAA may also be important for other therapeutic decisions, as anticoagulation in particular is a critical issue in CAA patients [2,3], especially as there are alternative treatment approaches like atrial appendage occlusion. In 2022, the Modified Boston Criteria were updated. Notably, white matter lesions as a neuroradiological criterion were added [4], which now allows for the diagnosis of possible CAA in the absence of hemorrhagic lesions. The diagnosis of probable CAA requires the presence of a lobar hemorrhagic lesion and the presence of a non-hemorrhagic lesion or another hemorrhagic lesion. Overall sensitivity and specificity could be increased to 74.5% and 95%, respectively. The diagnosis of CAA is a four-tiered system using a combination of clinical and neuroradiological parameters (Table 1). Since neuroimaging is a central part of the Boston Criteria, knowledge of the imaging findings associated with CAA and their correct application is essential. In this imaging review, typical imaging findings, which are part of the Boston Criteria 2.0 and other neuroradiological findings which were not included, along with a short explanation of their pathophysiological basis will be demonstrated.

## 2. Diagnosis

The highest category (definite CAA) can only be made postmortem since a brain autopsy is necessary. It requires the presence of an intracranial hemorrhage, transient focal neurological episodes, convexity subarachnoid hemorrhage or cognitive impairment or dementia. Of note, the histopathological manifestation of CAA demonstrated on brain autopsy must be severe (see Histopathological Findings).

The second highest category (probable CAA with supporting pathology) requires a brain biopsy with vessels in the specimen. It also requires the presence of an intracranial hemorrhage, transient focal neurological episodes, convexity subarachnoid hemorrhage or cognitive impairment or dementia. However, any degree of histopathological manifestation of CAA in the specimen is considered sufficient for the diagnosis of CAA.

In both categories, no age limits apply, although in the young, hereditary or iatrogenic forms of CAA should be considered and excluded [5].

Probable CAA diagnosis does not require histopathological support and can be made non-invasively in vivo. It requires a clinical presentation with spontaneous intracerebral hemorrhage, transient focal neurological episodes, convexity subarachnoid hemorrhage or cognitive impairment or dementia. In addition, the presence of a combination of two hemorrhagic lesions (intracerebral hemorrhage, cerebral microbleeds, cortical superficial siderosis, convexity subarachnoid hemorrhage) or the combination of one hemorrhagic lesion **and** one non-hemorrhagic lesion (enlarged perivascular spaces in the centrum semiovale or white matter hyperintensities in a multispot pattern) on MRI is required (Table 1).

Possible CAA differs from probable CAA only in terms of the requirement of the presence of only one hemorrhagic or non-hemorrhagic lesion.

In both probable and possible CAA, no other cause for hemorrhagic lesions must be identified, and no deep-seated hemorrhagic lesions must be present. Note that probable and possible CAA apply to patients over the age of 50 years as opposed to 55 years, as defined in the Modified Boston Criteria.

## 3. Histopathological Findings

**Figure 1:**
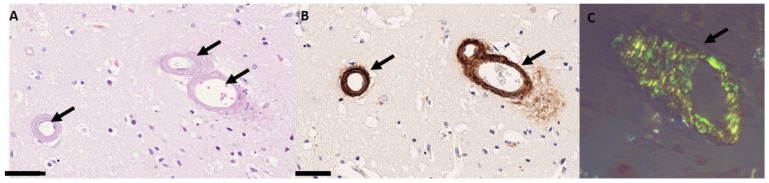
Histopathological findings in cerebral amyloid angiopathy. The cortical blood vessels are thickened and show hyalinosis of the vessel wall ((**A**), arrows). Immunohistochemical staining illustrates the deposition of beta-amyloid ((**B**), arrows) by brown staining in this case. The bar in the left lower corner represents 50 µm. (**C**) Congo red staining under polarization microscopy demonstrating a yellow-green birefringence (arrows, from [7]).

Cerebral amyloid angiopathy is caused by the accumulation of amyloid-β in cerebral and leptomeningeal vessel walls. On conventional histopathology, a thickened wall and narrowed lumen can be demonstrated (Figure 1A). Historically, Congo red staining was used to show a yellow-green birefringence which can be demonstrated under polarization microscopy (Figure 1C). Nowadays, immunohistochemistry with labeled antibodies against amyloid can specifically prove the presence of amyloid-β (Figure 1B). Histopathological changes are rated according to the Vonsattel rating scale which is a four-tiered scale (0: absent; 1: some amyloid deposits present, otherwise normal vessel; 2: replacement of the media by amyloid, wall is thicker than normal; 3: extensive amyloid deposition with focal wall fragmentation or double barreling of the vessel wall) [6]. Nowadays, however, given the advances in non-invasive diagnosis brain biopsy in the absence of intracranial hemorrhage, evacuation is rarely needed or performed.

## 4. Clinical Criteria

All diagnostic categories of the Boston Criteria 2.0 require the presence of an intracranial hemorrhage, transient focal neurological episodes, convexity subarachnoid hemorrhage or cognitive impairment or dementia.

Intracranial hemorrhages, namely lobar hemorrhages, cause apoplectic stroke-like symptoms sometimes accompanied by headaches and epileptic seizures (also see *Neuroradiological criteria, Hemorrhagic lesions, Lobar hemorrhages*).

Transient focal neurological episodes are short-lasting (<1 h), recurrent monomorphic episodes of neurological symptoms sometimes referred to as amyloid spells. They are characterized by typical positive (limb jerking, spreading paresthesia) or negative symptoms (sudden loss of motor, sensory or speech function) [8]. They are thought to be caused by cortical excitation induced by cortical superficial siderosis or subarachnoid hemorrhage. It can be challenging to differentiate TFNEs from a transient ischemic attack and other conditions mimicking short-lasting episodes of neurological impairment. TNFEs are more likely to exhibit migratory spread, affect sensation and recur in a stereotyped manner [9].

Cognitive impairment or dementia is an umbrella diagnosis not further specified in the Boston Criteria. In CAA, global cognition is frequently impaired, while the most commonly impaired domains are verbal and non-verbal IQ, executive functions and processing speed [10,11]. Since a pathological and clinical continuum between CAA and AD as the most frequent form of dementia in the elderly probably does exist [12], it can be challenging and at times impossible to detangle their respective contributions.

Neuroimaging with the strict application of the diagnostic criteria defined in the Boston Criteria paired with clinical reasoning can significantly contribute to obtaining a definite diagnosis in suspected cerebral amyloid angiopathy.

## 5. Neuroradiological Criteria

### 5.1. Hemorrhagic Lesions

#### 5.1.1. Lobar Hemorrhages

**Figure 2:**
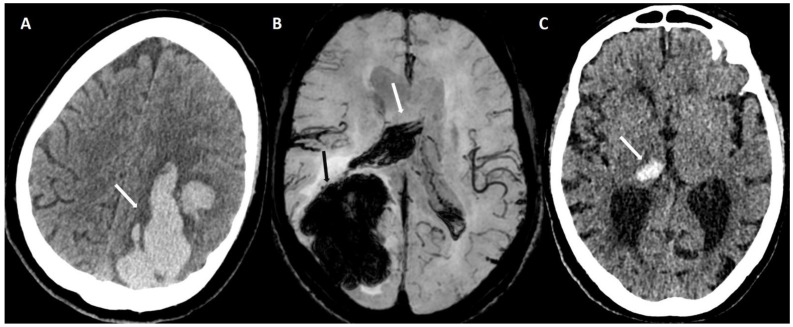
(**A**) Left parietal lobar hemorrhage (white arrow) on non-enhanced CT (100 kV, 596 mAs, slice thickness 1 mm), (**B**) right occipital lobar hemorrhage (black arrow) with intraventricular extension (white arrow) on axial venBOLD MRI (1.5 T, TE = 49 ms, TR = 34 ms, 10 mm MinIP). (**C**) Deep-seated right sided thalamic hemorrhage (white arrow) on non-enhanced CT (100 kV, 394 mAs, slice thickness 1 mm).

Lobar hemorrhages are located in the frontal, parietal, occipital, temporal or insular lobe of the brain (Figure 2A,B). Deep-seated hemorrhages in the basal ganglia, thalami or brain stem are not typical (Figure 2C) and are associated with arterial hypertension [13]. Other causes of lobar hemorrhages (aneurysm, arterio-venous malformations, vein thrombosis, cavernomas, tumor) can be detected with additional neuroradiological work-up such as a CT angiography [14], MRI or digital subtraction angiography. Lobar hemorrhages appear as a lobulated or bizarre hyperdense structure on CT with mass effect and effacement of the adjacent sulci sometimes accompanied by perifocal edema.

#### 5.1.2. Multiple Cerebral Microbleeds

**Figure 3:**
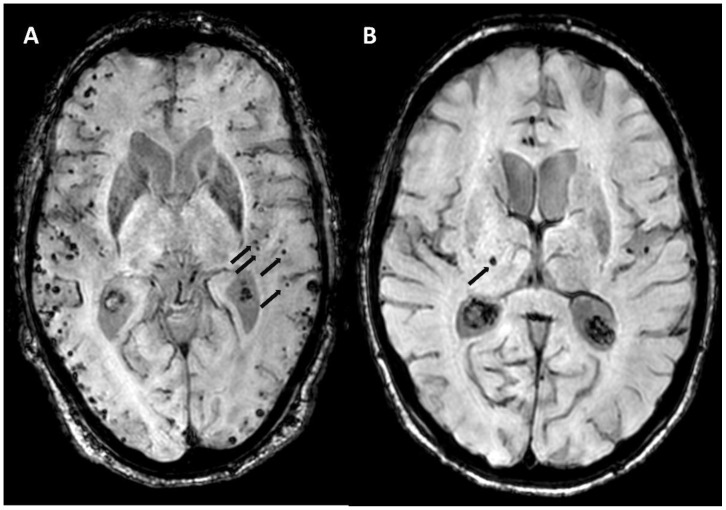
(**A**) Multiple lobar cerebral microbleeds (black arrows; not all microbleeds are marked). (**B**) Single deep-seated thalamic CMB (black arrow) on venBOLD MRI (3T, TE = 25 ms, TR = 18 ms, slice thickness 1 mm).

Cerebral microbleeds (CMBs) are small (2–10 mm) round or ovoid homogeneously hypointense structures on susceptibility-based sequences (T2* or susceptibility-weighted imaging, SWI). Multiple (≥2), lobar, cortical or subcortical CMBs are typical for but not exclusively found in CAA (Figure 3A). A certain preference for posterior brain regions has been described [15]. Like lobar hemorrhages, deep-seated CMBs are associated with arterial hypertension (Figure 3B). Other causes and mimics of multiple parenchymal susceptibility artifacts (diffuse axonal injury, status post-heart–lung machine [16], brain irradiation, hemorrhagic metastasis, vasculitis, fat embolism) can be excluded with thorough history taking and a dedicated neuroradiological work-up. In general, the number of CMBs is positively correlated with an increased risk of intracranial hemorrhage [17].

If the diagnosis of CAA is made non-invasively, i.e., without histopathological confirmation, no hemorrhagic lesions (CMB or macrohemorrhages) in deep brain structures must be present. However, a phenotype with mixed location hemorrhages with features of CAA and deep-seated hemorrhagic lesions exists [18,19] but is not covered by the Boston Criteria. It is unclear if in these patients both entities are present, or if it is an extreme form of hypertensive arteriopathy.

#### 5.1.3. Convexity Subarachnoid Hemorrhage

**Figure 4:**
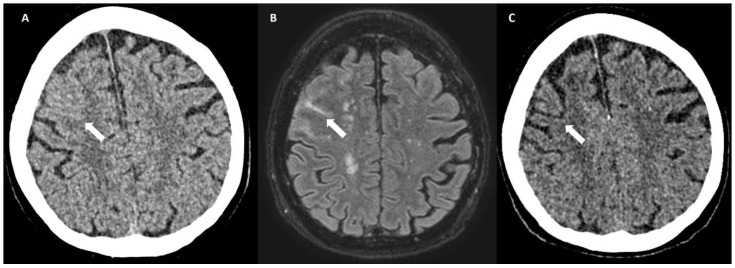
Right frontal subarachnoid hemorrhage on non-enhanced CT (**A**), 100 kV, 394 mAs, slice thickness 1 mm) and FLAIR MRI (**B**), 1.5 T, TE = 319 ms, TR = 4800 ms, TI = 1660 ms, slice thickness 3 mm) visible as the hyperdense/hyperintense effacement of the sulcus (A and B, white arrows). The control image ((**C**), non-enhanced CT, 100 kV, 394 mAs, slice thickness 1 mm) demonstrates the clearing of the sulcus (arrow).

Due to the involvement of the leptomeningeal vessels, recurrent subarachnoid hemorrhages (SAHs) can occur in CAA. They are typically located at the convexity (Figure 4) but not in the basal cisterns as typical aneurysmatic subarachnoid hemorrhages are [20,21]. They are best appreciated on CT as the hyperdense effacement of the sulci, or on MRI, FLAIR demonstrates hyperintense signals in these areas consistent with acute hemorrhage.

#### 5.1.4. Cortical Superficial Siderosis

**Figure 5:**
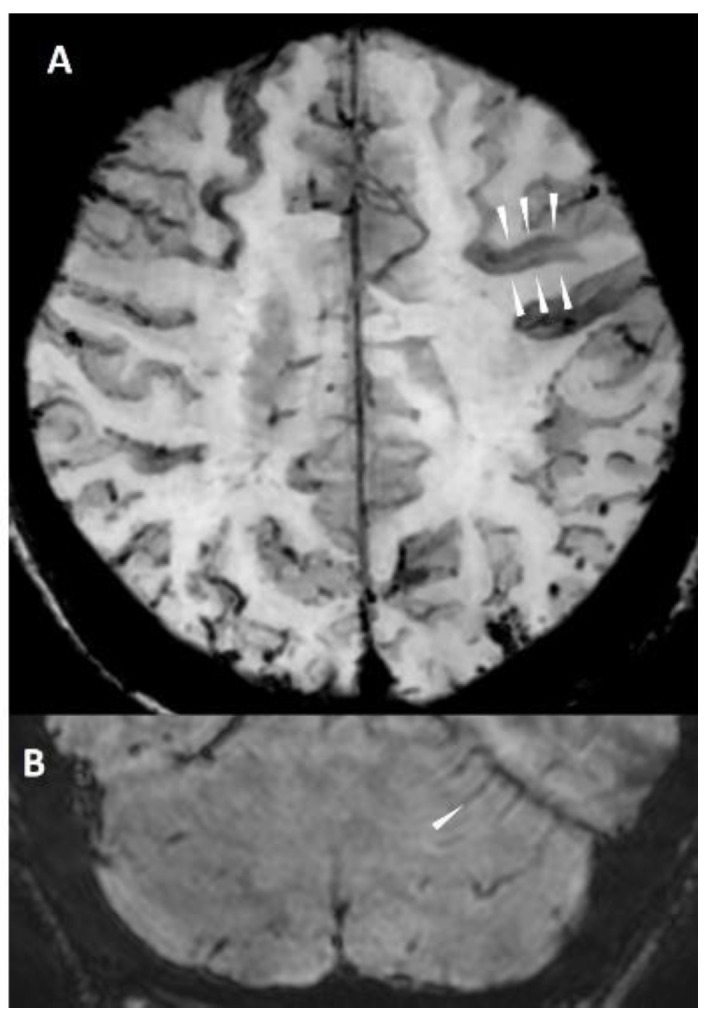
Cortical superficial siderosis. The coating of both surfaces of the sulcus can be appreciated ((**A**), white arrowheads) on axial venBOLD MRI (3T, TE = 25 ms, TR = 18 ms, 10 mm MinIP). Infratentorial siderosis along the folia of the cerebellum ((**B**), white arrowhead) on axial venBOLD MRI (1.5 T, TE = 49 ms, TR = 34 ms, slice thickness 2.2 mm).

A hypointense curvilinear coating following the surface of the cortex is called cortical superficial siderosis (cSS) [22]. Histologically, hemosiderin laden macrophages can be found. It is likely but not finally proven that they are the sequela of an acute subarachnoid hemorrhage. A focal (<4 sulci) and disseminated (≥4 sulci) form can be differentiated [3]. They are thought to be the origin of TFNEs or amyloid spells. Cortical superficial siderosis is considered a highly specific marker for CAA and is associated with an increased risk for future or recurrent intracranial hemorrhages [23]. Infratentorial cSS is associated with severe CAA [24] (Figure 5B).

### 5.2. Non-Hemorrhagic Lesions

#### 5.2.1. Enlarged Perivascular Spaces

**Figure 6:**
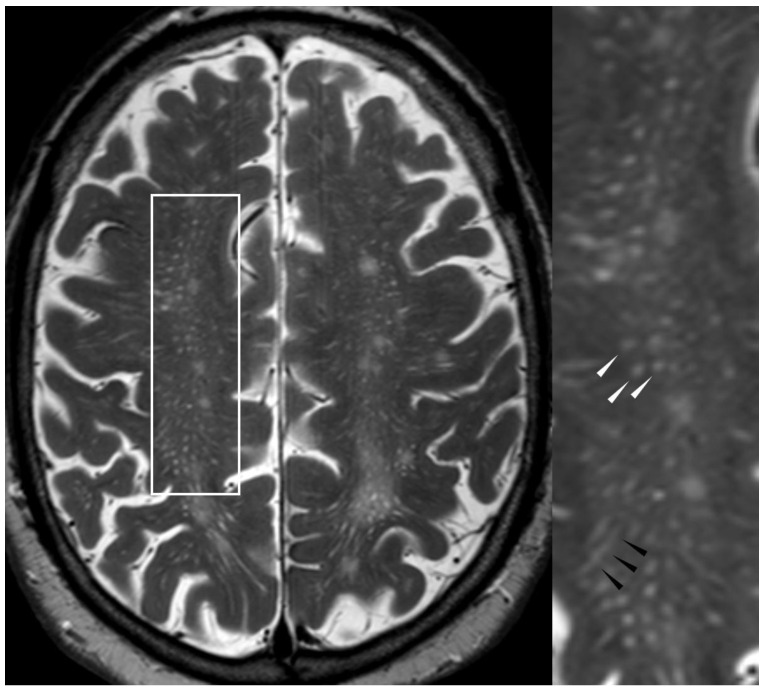
Enlarged perivascular spaces in the centrum semiovale on axial T2w (3 T, TE = 80 ms, TR = 5500 ms, slice thickness 2 mm). Multiple EPVSs appear as round structures if the plane of acquisition runs perpendicular to the course of the perivascular space (white arrowheads) and as a linear structure when it runs along the course of the perivascular space (black arrowheads).

Perivascular spaces are physiological spaces filled with cerebrospinal fluid surrounding penetrating arteries. In CAA, they are enlarged probably due to impaired resorption. They are visible on T2w images as round or linear hyperintense structures depending on the plane of acquisition (Figure 6). Typically, there is only a very fine, if any, rim of hyperintensity surrounding enlarged perivascular spaces (EPVSs) but no overt gliosis. In order to fulfill this newly added criterion, more than 20 EPVSs in the centrum semiovale per hemisphere must be present [25]. EPVSs on the level of the basal ganglia are not typical for CAA but are associated with arterial hypertension.

#### 5.2.2. Subcortical White Matter Lesions

**Figure 7:**
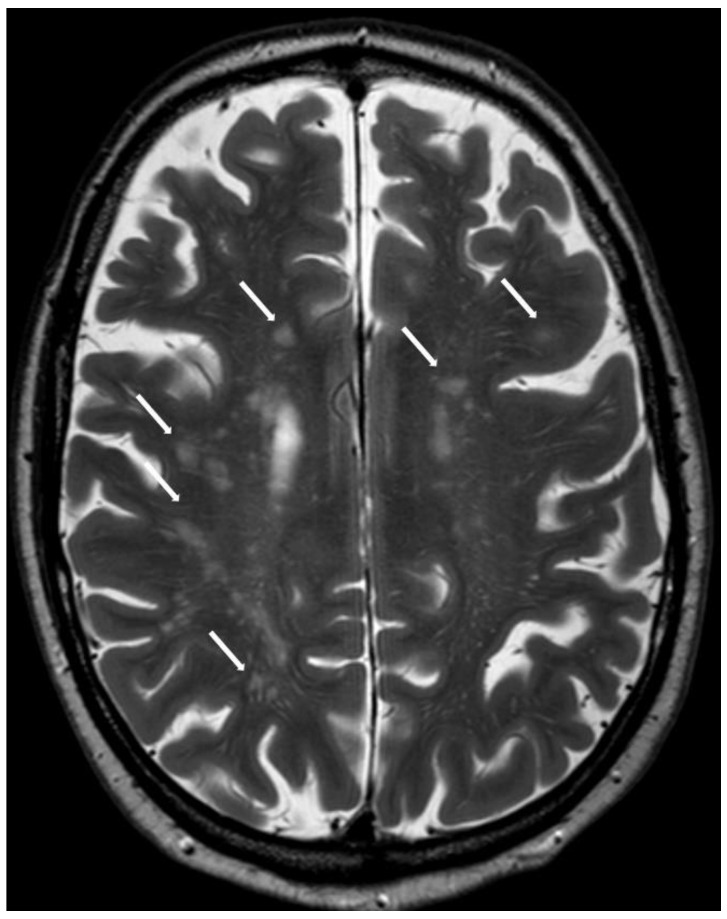
Multiple subcortical white matter lesions referred to as the multispot pattern (white arrows; not all lesions are marked) on axial T2w (3 T, TE = 80 ms, TR = 5500 ms, 2 mm slice thickness).

White matter lesions (WMLs) are a common finding in the elderly. The classical description distinguishes periventricular WMLs from WMLs in the deep white matter, the latter being more relevant and thought to represent gliosis from small vessel ischemia [26]. A new anatomic location in the subcortical region was added to the Boston Criteria 2.0. In order to fulfill this white matter criterion, more than 10 subcortical lesions (not counting periventricular lesions or lesions in the deep white matter) must be present. This pattern is called the multispot pattern [27] (Figure 7).

It has to be emphasized that owing to the added white matter characteristics, possible CAA can now be diagnosed even in the absence of hemorrhagic lesions. However, sensitivity and specificity are lower as CAA may only have a minor contribution to the clinical picture [28].

A recent systematic review determined the prevalence of these imaging and clinical markers. The most prevalent imaging markers were enlarged perivascular spaces in the centrum semiovale (56%), CMBs (52%), cSS (49%) and ICH (44%). Clinically, 50% had dementia, and 48% had TFNEs in a hospital setting [29].

## 6. Neuroradiological Findings That Are Not Part of the Boston Criteria

Some typical imaging findings of CAA are not part of the Boston Criteria. While some of them are the target of ongoing research, others are already incorporated in diagnostic criteria.

### 6.1. Finger-like Projections

**Figure 8:**
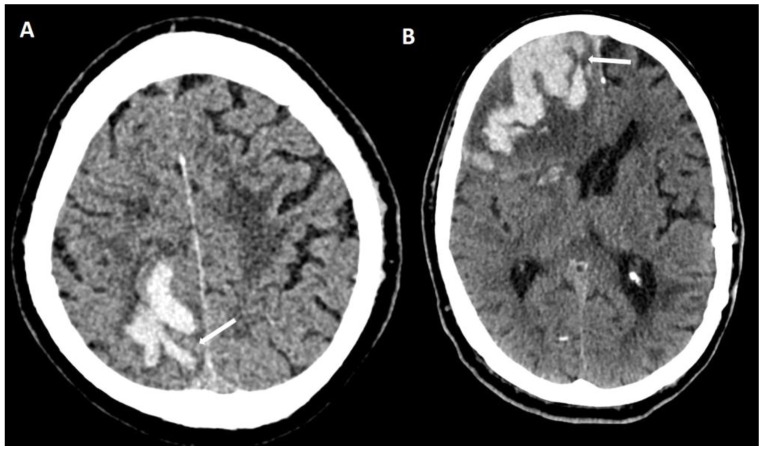
Typical examples of finger-like projections (white arrows) on non-enhanced CT ((**A**) 100 kV, 394 mAs, slice thickness 1 mm; (**B**) 120 kV, 320 mAs, slice thickness 1 mm).

**Figure 9:**
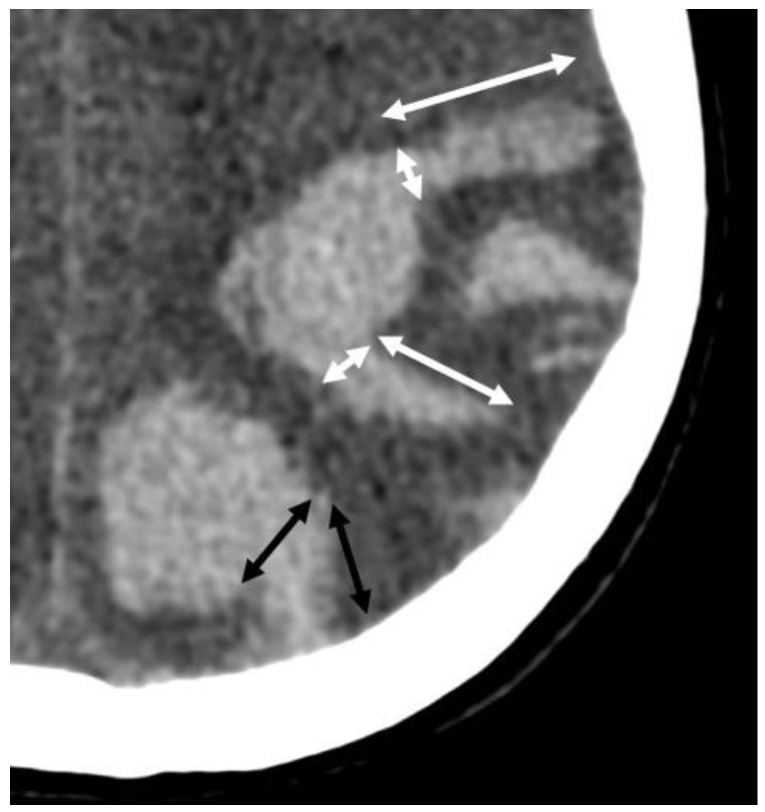
Finger-like projections on non-enhanced CT (slice thickness 1 mm, 100 kV, 394 mAs). The length of the projection exceeds its width at the base where it emerges from the main hemorrhage (white double arrows). The lobulation of a hemorrhage with the width of the base approximately the same as its length (black double arrow).

**Figure 10:**
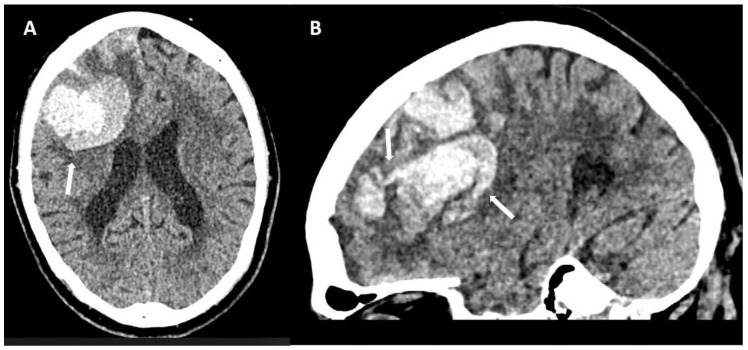
Demonstrating the need for 3D reconstruction. No finger-like projection can be spotted on non-enhanced CT (100 kV, 394 mAs, slice thickness 1 mm) in the axial plane, only a lobulation ((**A**), arrow). The sagittal reconstruction demonstrates at least two finger-like projections ((**B**), arrows).

This term refers to a finger-like projection out of a main hemorrhage (Figure 8). By definition, the length of the base of the projection must exceed its length (Figure 9). For optimal detection, a hemorrhage should be examined in all three planes using multiplanar rendering [30,31] (Figure 10). If the base of the projection is broader than its length, it is called a lobulation and not considered typical for CAA. It is mainly used on non-enhanced CT but has also been described on MRI [30].

### 6.2. Subarachnoid Extension of Lobar Hemorrhage

**Figure 11:**
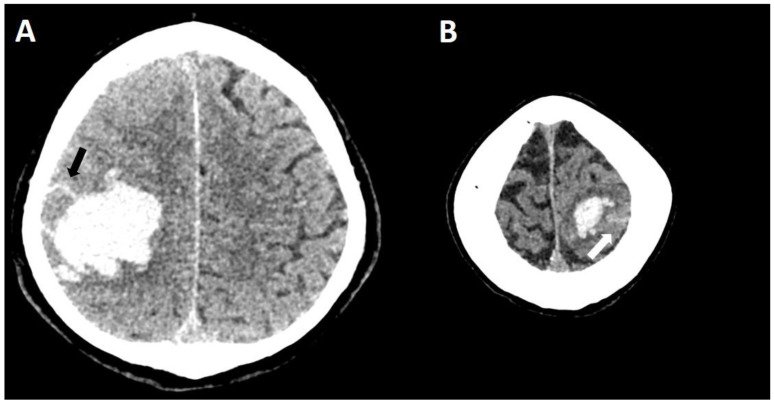
Subarachnoid extension of lobar hemorrhage demonstrated on non-enhanced CT ((**A**): 100 kV, 596 mAs, slice thickness 1 mm; (**B**): 120 kV, 320 mAs, slice thickness 1 mm). Leakage of blood into subarachnoid space effacing adjacent sulci (arrows).

If a lobar hemorrhage has an extension into the subarachnoid space, i.e., if an adjacent SAH is present, this is considered a marker for CAA [31,32] (Figure 11). Subarachnoid extension is probable due to the spatial proximity of the origins of these hemorrhages to the subarachnoid space. Further, since CAA predominantly affects the subarachnoid and cortical vessels, it is conceivable that leptomeningeal vessels rupture directly into the subarachnoid space.

Both CT imaging criteria (finger-like projections and subarachnoid extension) are incorporated in the Edinburgh criteria, which additionally include the APOE genotype [33].

These criteria allowed for an excellent discrimination between CAA- and non-CAA-related hemorrhages. Note that the Edinburgh criteria were validated in patients who died due to an intracranial hemorrhage. Whether these criteria apply to smaller lobar hemorrhages remains to be demonstrated [31].

### 6.3. DWI Lesions

**Figure 12:**
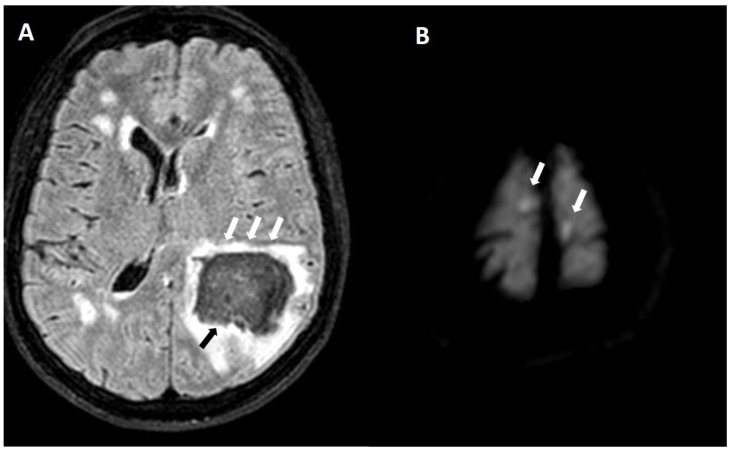
(**A**) Left parietal lobar hemorrhage (black arrow) with adjacent edema (white arrows) on axial FLAIR (1.5 T, TE = 385 ms, TR = 7000 ms, TI = 2050 ms, slice thickness 3 mm). (**B**) Bilateral cortical remote diffusion-weighted imaging lesions (arrows) on axial DWI (1.5 T, TE = 57 ms, TR = 2600 ms, b = 1000, slice thickness 5 mm).

Acute DWI lesions remote from the site of hemorrhage (remote DWI lesions, RDWILs) are observed in patients with acute ICH with a prevalence of ~30% within 3 days of stroke onset. They are more frequently located in lobar regions than in deep brain structures and are often multiple (Figure 12). Pathophysiologically, they may be the consequence of acutely raised intracranial pressure or the impairment of cerebral autoregulation [34,35].

These imaging features (finger-like projections, subarachnoid extension, RDWILs) are currently the target of evaluation in patients who survived lobar hemorrhages and in whom CAA is suspected [36].

### 6.4. Lobar Lacunes

**Figure 13:**
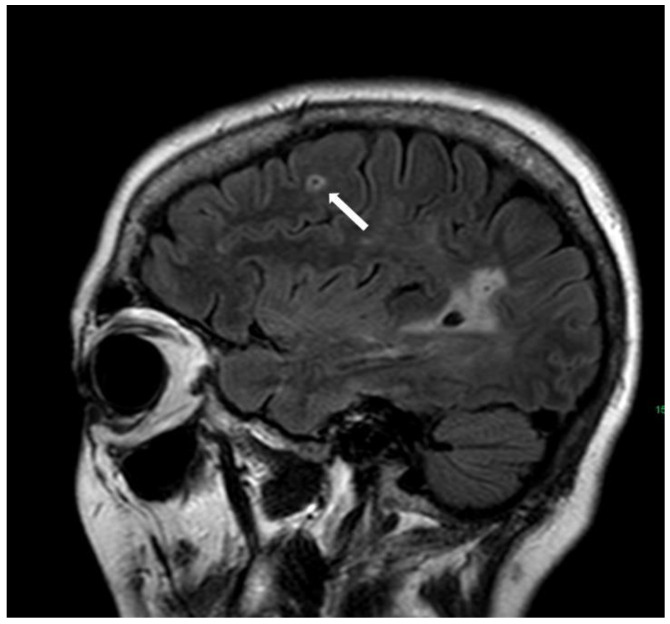
A lobar lacune in the frontal subcortical white matter with surrounding gliosis (arrow) on sagittal FLAIR (3 T, TE = 160 ms, TR = 12,000 ms, TI = 2850 ms, slice thickness 3 mm).

A lobar lacune is a residual cavitation consistent with a previous infarct of a penetrating artery in the white matter [37]. They present as small (3–15 mm in size) fluid-filled spaces surrounded by a rim of gliosis. Like lobar hemorrhages, they are located in the lobar position (Figure 13) and not in deep-seated structures (thalami, basal ganglia or the brain stem).

### 6.5. Posterior White Matter Changes

**Figure 14:**
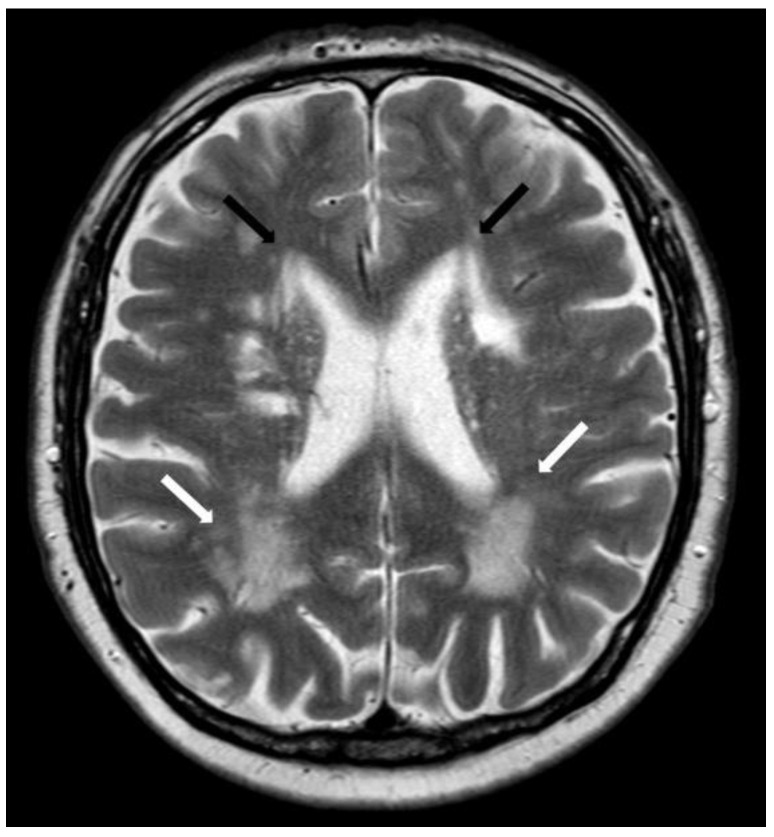
Posterior dominance of periventricular white matter lesions on axial T2w (3 T, TE = 80 ms, TR = 5500 ms, slice thickness 2 mm) with significantly fewer white matter lesions at frontal horns (black arrows) than posterior horns (white arrows).

The posterior dominance of periventricular white matter lesions (Figure 14) can be indicative of CAA but is only a weak predictor [27]. Periventricular white matter lesions are thought to result from a range of pathophysiological processes [38]. In CAA, the posterior regions of the brain are preferentially involved [39], potentially resulting in the pattern of the posterior dominance of periventricular white matter lesions.

### 6.6. Cortical Calcifications

**Figure 15:**
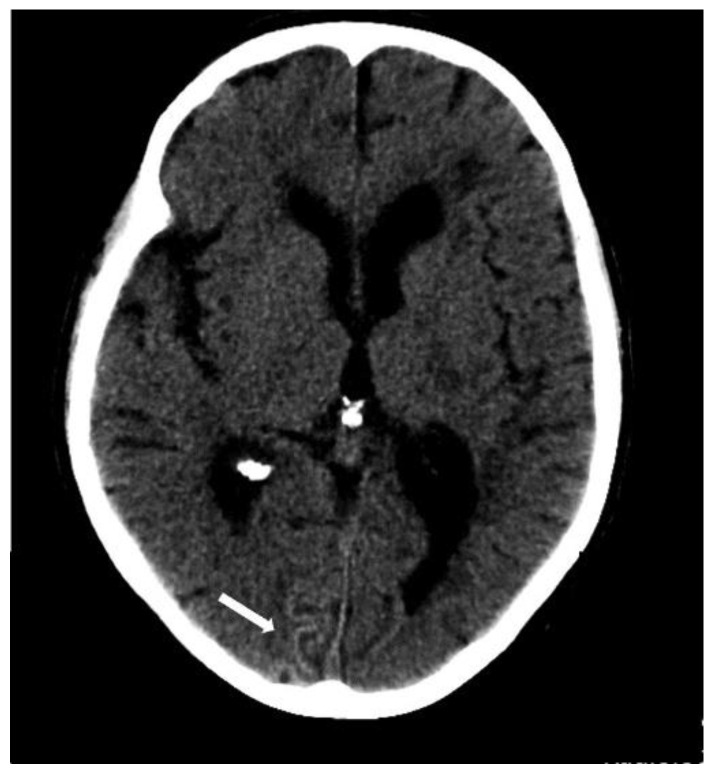
Gyriform calcification of right occipital cortex (arrow) on non-enhanced CT (image courtesy I. Rasing, Leiden/The Netherlands).

Gyriform calcifications of the cortex (Figure 15) typically seen in the occipital lobe were described in hereditary forms of CAA but are rare in spontaneous CAA. They are thought to represent calcification and iron accumulation in the wall of arteries in regions most susceptible to CAA-related disease and could indicate advanced CAA [40].

## 7. Discussion

The Boston Criteria v2.0 allow for the diagnosis of CAA in symptomatic patients (intracerebral hemorrhage, convexity subarachnoid hemorrhage, transient focal neurological episodes, dementia) with a combination of hemorrhagic (intracerebral hemorrhage, cerebral microbleeds, cortical superficial siderosis, convexity subarachnoid hemorrhage) and non-hemorrhagic (enlarged perivascular spaces in the centrum semiovale or white matter hyperintensities in a multispot pattern) imaging markers. Finger-like projections and the subarachnoid extension of lobar hemorrhages are part of the Edinburgh criteria and can be appreciated on CT and MRI. Several other imaging markers are suggestive (posterior WML dominance, lobar lacunes, cortical calcification) but either rare, have a low predictive value or are mostly seen in hereditary CAA.

Brain biopsy demonstrating amyloid deposition in cortical and leptomeningeal vessels is rarely needed. However, in certain constellations when CAA cannot be diagnosed but is highly suspected, other less invasively obtainable biomarkers such as CSF [41,42] and the assessment of amyloids and tau or amyloid PET [43] can be helpful.

## 8. Conclusions

Knowledge and the correct application of the Boston Criteria v2.0 and the identification of their hemorrhagic and non-hemorrhagic imaging markers and the presence of other imaging markers of CAA can help to achieve a prompt and reliable diagnosis of cerebral amyloid angiopathy.

## Figures and Tables

**Table 1:** The Boston Criteria for the diagnosis of cerebral amyloid angiopathy—version 2.0.

**Definite CAA**
Full brain postmortem demonstrating the following:
Spontaneous intracerebral hemorrhage, transient focal neurological episodes, convexity subarachnoid hemorrhage or cognitive impairment or dementia.
Severe CAA with vasculopathy.
Absence of other diagnostic lesions.
**Probable CAA with supporting pathology**
Clinical data and pathological tissue demonstrating the following:
Presentation with spontaneous intracerebral hemorrhage, transient focal neurological episodes, convexity subarachnoid hemorrhage or cognitive impairment or dementia.
Some degree of CAA in specimens.
Absence of other diagnostic lesions.
**Probable CAA**
For patients >50 years, clinical data and MRI demonstrating the following:
Presentation with spontaneous intracerebral hemorrhage, transient focal neurological episodes, convexity subarachnoid hemorrhage or cognitive impairment or dementia.
At least two of the following lobar hemorrhagic lesions on T2*-weighted MRI: intracerebral hemorrhage, cerebral microbleeds, cortical superficial siderosis or convexity subarachnoid hemorrhage.
OR
One lobar hemorrhagic lesion plus one white matter feature (enlarged perivascular spaces in the centrum semiovale or white matter hyperintensities in a multispot pattern).
Absence of deep hemorrhagic lesions.
Absence of other causes of hemorrhagic lesions.
**Possible CAA**
For patients > 50 years, clinical data and MRI demonstrating the following:
Presentation with spontaneous intracerebral hemorrhage, transient focal neurological episodes, convexity subarachnoid hemorrhage or cognitive impairment or dementia.
Absence of other causes of hemorrhagic lesions.
One lobar hemorrhagic lesion on T2*-weighted MRI: intracerebral hemorrhage, cerebral microbleeds, cortical superficial siderosis or convexity subarachnoid hemorrhage.
OR
One white matter feature (enlarged perivascular spaces in the centrum semiovale or white matter hyperintensities in a multispot pattern).
Absence of deep hemorrhagic lesions.
Absence of other causes of hemorrhagic lesions.

## Data Availability

All relevant data are included in this manuscript.
